# A novel spinal brace in management of scoliosis due to cerebral palsy. Radiological and subjective clinical results after at least one year of treatment

**DOI:** 10.1186/1748-7161-8-S2-O49

**Published:** 2013-09-18

**Authors:** Ichiro Kajiura, Yu Moriguchi, Yoshihiro Matsui, Tokimitsu Morimoto, Yohei Matsuo, Motoki Iwasaki, Tsunehiko Suzuki

**Affiliations:** 1 Osaka Developmental Rehabilitation Center Minami Osaka Hospital for Handicapped Children, Osaka, Japan

## Background

Severe scoliosis in patients with cerebral palsy (CP) causes difficulty in sitting balance and creates increased nursing demands. Surgical stabilization has proven to be a valuable method to stop the progression of scoliosis [1]. However, the complication rate after such surgery is substantial[2]. Additionally, many patients with quadriplegia and large curvatures of the spine have impaired general health, epilepsy and reduced respiratory capacity, making them poor candidates for major surgery like spine fusion. Therefore, other treatment alternatives should be available. We have recently developed a spinal brace named Dynamic Spinal Brace (DSB), which is a custom-molded, polycarbonate orthosis characterized by lightness and flexibility. Unlike the other underarm orthoses, DSB does not fix the pelvic girdle rigidly and, thus, potentially contributes to good compliance with bracing. 

## Purpose

The purpose of this study was to examine the efficacy of DSB for the management of scoliosis in CP patients. 

## Methods

A total of 151 patients with CP and scoliosis have been treated by DSB (age: 14.3 ± 8.2 years). The mean follow-up period was 33.9 ± 11.9 months. Periodic x-ray tests in the sitting position were performed in order to evaluate in-brace correction and curve progression. Questionnaires of the caretakers were performed to evaluate activities of daily living (ADL) of the patients.

## Results

Cobb angle with and without DSB were 42.1 ±30.2 and 60.0 ± 33.7 degrees, respectively. Initial in-brace curve correction was 17.8 ± 11.6 degrees. The curve progression per year was 3.8 ± 7.0 degrees. Initial in-brace correction was negatively correlated with curve progression (p<0.05). In all, 84.9% of the caretakers reported that DSB enhanced sitting stability. Only two patients dropped out of the study. 

## Conclusions and discussion

Many of the spinal braces designed for idiopathic scoliosis do not necessarily match the needs of CP patients with more complicated medical conditions. DSB is specifically designed for CP patients and, therefore, showed good compliance, moderate in-brace correction equivalent to precedent braces[3], and potential contributions to improvement of the ADL of the patients. Although the preventive impact on curve progression remains to be elucidated by the longer follow-up, DSB could be an option for the management of scoliosis in CP patients. 
